# Initiatives to increase breast and cervical cancer–related knowledge, screening, and health behaviours among Black women

**DOI:** 10.17269/s41997-024-00953-y

**Published:** 2024-10-22

**Authors:** Camille Williams, Elaine Goulbourne, Elijah Gyansa, Ayan Hashi, Ielaf Khalil, Rumaisa Khan, Patricia Rabel-Jeudy, Ruth Heisey, Aisha Lofters

**Affiliations:** 1 The Peter Gilgan Centre for Women’s Cancers, Women’s College Hospital, Toronto, ON Canada; 2 Faculty of Arts & Sciences, University of Toronto, Toronto, ON Canada; 3 Sinai Health, Toronto, ON Canada; 4 Department of Family and Community Medicine, University of Toronto, Toronto, ON Canada

**Keywords:** Health promotion, Early detection of cancer, Breast cancer, Gynecologic disease, Culturally congruent care, Health equity, Black people, Female, Ontario, Promotion de la santé, Dépistage précoce du cancer, Cancer du sein, Maladies de l’appareil génital féminin, Soins adaptés sur le plan culturel, Équité en santé, Personnes noires, Femmes, Ontario

## Abstract

**Setting:**

In Canada, racialized and immigrant women are typically under-screened for breast and cervical cancer. Under-screening is linked to numerous barriers to access, including lack of awareness, fear of pain, the stigma of cancer, socio-cultural factors like language, and various socio-economic factors. To address these barriers, our team developed a series of initiatives to promote awareness of breast and cervical health among Black women.

**Intervention:**

Building on the development of a breast cancer resource hub for Black women, and in partnership with relevant community organizations, we implemented a series of virtual educational and cancer screening events (two of each thus far). Both event series were targeted towards Black women and tailored to their needs.

**Outcomes:**

Each educational event attracted more than 450 attendees and had average attendance times > 1 h. Most (> 87%) survey respondents agreed that an event specifically for Black women helped them feel supported. The 2022 and 2023 screening events provided breast and/or cervical cancer screening for 46 and 48 women, respectively. In both years, most women (> 90% of question respondents) noted that they were (extremely) likely to go for a mammogram or Pap test when next due.

**Implications:**

Both event series provided targeted opportunities for Black women to learn about prevention, risk factors, resources, and screening related to women’s cancers. It is possible that, over time, such culturally tailored events can reduce or remove the stigmas associated with cancer and decrease differences in cancer-related knowledge and behaviours between racialized and non-racialized groups.

**Supplementary Information:**

The online version contains supplementary material available at 10.17269/s41997-024-00953-y.

## Résumé

**Lieu:**

Au Canada, le dépistage des cancers du sein et du col de l’utérus ne rejoint généralement pas de façon constante les femmes racisées et immigrantes. Ce sous-dépistage est lié à de nombreux obstacles à l’accès, dont le manque de connaissances, la peur de la douleur, la stigmatisation du cancer, les facteurs socioculturels comme la langue, et divers facteurs socioéconomiques. Pour aborder ces obstacles, notre équipe a mis au point une série d’initiatives de sensibilisation des femmes noires à la santé du sein et du col de l’utérus.

**Intervention:**

Après avoir établi un centre de ressources sur le cancer du sein pour les femmes noires, nous avons mis au point, en partenariat avec des organismes associatifs intéressés, deux activités virtuelles d’éducation au cancer et deux activités de dépistage du cancer (pour l’instant). Les deux séries s’adressaient aux femmes noires et étaient adaptées à leurs besoins.

**Résultats:**

Chaque activité éducative a attiré plus de 450 participantes, qui y ont assisté pendant plus d’une heure en moyenne. La plupart (> 87 %) des répondantes au sondage ont dit s’être senties soutenues par une activité s’adressant spécifiquement aux femmes noires. Dans le cadre des activités de dépistage menées en 2022 et en 2023, 46 et 48 femmes, respectivement, ont subi des tests de dépistage du cancer du sein ou du col de l’utérus. Les deux années, la plupart des femmes (> 90 % de celles ayant répondu à la question) ont dit qu’elles passeraient (très) probablement une mammographie ou un frottis à leur prochaine échéance.

**Conséquences:**

Les deux séries d’activités ont représenté des occasions pour les femmes noires de se renseigner sur la prévention, les facteurs de risque, les ressources et le dépistage liés aux cancers féminins. Il est possible qu’avec le temps, de telles activités culturellement adaptées atténuent ou suppriment la stigmatisation associée au cancer et réduisent l’écart entre les groupes racisés et non racisés dans les connaissances et les comportements liés au cancer.

## Introduction

Attention to anti-Black racism as a determinant of health and as a system of inequities in power has highlighted the extent to which Black people’s experiences of care or utilization of services for various health conditions fall below targets (Public Health Agency of Canada, 2020). One area of concern is the cancer continuum. Particular focus on Black women’s experiences of cancer care arose from the 2022 American Cancer Society finding that Black women in the United States are 41% and 65% more likely to die from breast and cervical cancer than white women (Giaquinto et al., 2022). These figures are alarming, in part, because both these cancers can be detected early with screening and self-reported screening rates are similar for both Black and white women (Giaquinto et al., 2022). Understandably, Black women in Canada want to know if their risks are also increased.

In Canada, reduction in the burden of breast and cervical cancers has been attributed, in part, to provincially organized screening programs (Canadian Cancer Statistics Advisory Committee in collaboration with the Canadian Cancer Society, Statistics Canada, and the Public Health Agency of Canada, 2021). Unfortunately, it is known that uptake of breast and cervical screening is uneven across the population. Research points to several sociodemographic characteristics that are associated with being never-screened or under-screened for breast and cervical cancers. They include being a recent immigrant, living with low income, and being unattached to a primary care provider, among others (Blackwell et al., 2008; Borkhoff et al., 2013; Evans et al., 2005; Health Quality Ontario, 2018; Lofters et al., 2014, 2019, 2018; Louwman et al., 2007). Given the large proportion of immigrants to Canada who would be considered a “visible minority” (Statistics Canada, 2022), it is important to understand whether and how race interacts with cancer screening behaviour and cancer-related outcomes for women in Canada.

In a scoping review of breast and cervical cancer studies focusing on Black women in Canada, the authors noted that the data available—academic and grey literature from 2003 to October 2018—supported the conclusion that women from sub-Saharan Africa tend to have lower screening rates while those from the Caribbean or Latin America have screening rates comparable to the general population (Nnorom et al., 2021). However, none of the reviewed studies reported the prevalence or mortality rates of cervical or breast cancer for Black women, precluding definitive conclusions about Black women’s risks. A later report examining cancer incidence and mortality rates by ethnicity demonstrated that cervical cancer was among the top ten cancers only for those of Caribbean and East Asian ethnicities (Hwee & Bougie, 2021). When examining the distribution of cancer deaths, cervical cancer was in the top ten cancers only for those of Caribbean ethnicity. This report also showed that the percentages of African and Caribbean women who died from breast cancer were 20.7% and 21.7% compared to 14.1% for women of European ethnicity. A more recent Statistics Canada report of mortality inequalities for Black adults in Canada using census data from three cohorts between 2001 and 2011 with follow-up data to the end of 2019 (Tjepkema et al., 2023) found that a higher percentage of Black women died from breast cancer (7.6%) than white women (4.6%). Deaths from cervical cancer were not reported.

These reports suggest that, like trends in the USA, there are social and healthcare inequities leading to higher cancer mortality for Black women in Canada; and, at least for some Black women, this could be related to lower screening uptake. However, Canadian data are often limited by lack of information on racialized identities, the use of rough proxies for racialization like ethnicity and immigration status, or small data sets where respondents report race but which present challenges for analysis and statistical inference due to their size (Public Health Agency of Canada, 2020). Although Canadian cancer surveillance statistics do not as clearly support the need for mechanisms and incentives that provide equitable cancer care for Black people as those from the USA, we believe that a proactive approach to developing services and materials that are tailored to and empowering for Black women to increase uptake of breast and cervical cancer screening is sensible while supporting data are being established.

## Background

Moved by the need for tailored approaches to cancer care for Black women, Women’s College Hospital (WCH) launched an online resource hub called Every Breast Counts (EBC) in 2022. EBC was created to address the gap in breast cancer information and support experienced by Black women. It was co-created by community partners and members of the Peter Gilgan Centre for Women’s Cancers (henceforth “The Centre”) at WCH, in partnership with the Canadian Cancer Society (CCS). EBC provides empowering and action-oriented messaging for Black women^1^ regarding breast health and breast cancer care (Hashi et al., 2023). The experience of co-creating this resource acted as a catalyst to develop two subsequent initiatives to help Black women access cancer information and cancer screening: a virtual educational event and a screening event. We describe these interventions in detail.

### Collaboration with community partners

Representatives from The Centre, WCH, community organizations, and/or individuals with lived experiences partnered to develop the interventions and contribute to their execution. All partner organizations serve the Black community and/or are led by individuals who have lived experiences of being racialized, navigating the Ontario healthcare system, or navigating cancer care. Staff from The Centre took the lead on developing the concepts for these events and then invited various community organizations to work with us to bring them to fruition. Interested organizations assigned 1–2 representatives (staff or volunteers) to join each planning team. Centre staff developed a project charter, including timeline and budget, and assumed all other responsibilities for project management such as scheduling and facilitating regular planning meetings. One-hour planning meetings were held biweekly in the early phases of planning and then moved to monthly or as needed during execution. Community partners and other relevant WCH staff were invited to all planning team meetings. These meetings were a collaborative space to provide feedback on the initial ideas of the project charter, brainstorm new ideas for the events, and share resources to achieve project goals.

For the educational events, we partnered with The Olive Branch of Hope and Rethink Breast Cancer. For the screening events, we partnered with The Olive Branch of Hope, TAIBU Community Health Centre, and Women’s Health in Women’s Hands Community Health Centre.

### Methods

The information and insights shared below were extracted from various program metrics and evaluations of the initiatives described (see Online Resource 1: Program Evaluation of Educational and Screening Events). Additionally, after each event, we conducted a debrief meeting. Notes and/or transcripts from these meetings were reviewed to identify challenges, lessons learned, and opportunities for improvement. These were presented to each planning team and further revised, if necessary.

## Intervention 1: Breast/Best Health for Black Women (BHFBW)

### Design and development

BHFBW is a national, free, virtual event—we have conducted two events thus far. The goals were to (1) empower Black women with breast health and wellness information tailored specifically to them and operationalize the mantra of “for us, by us”; (2) increase knowledge and awareness of risk factors, prevention, screening, and early detection; (3) provide an opportunity to learn through digital stories created by Black women with lived experiences of cancer; and (4) share educational and community resources available for Black women. Online Resource 1 includes the logic model for these events: a high-level overview of inputs, activities, audience, outputs, and outcomes. We define *outputs* as the quantified products or representations of the activities involved with delivering the intervention and *outcomes* as the changes in knowledge, awareness, opinions, behaviours, and skills that result from the intervention (Ontario Agency for Health Protection and Promotion (Public Health Ontario) et al., 2016). Table 1 links the barriers to cancer screening and elements of BHFBW that were designed to address these barriers—guided by recommendations from the Community Preventive Services Task Force for increasing cancer screening (Guide to Community Preventive Services, 2023).

**Table 1:** Intervention elements that addressed known barriers to screening for Black women

Barriers to screening	Elements of BHFBW events	Elements of the screening events
Lack of awareness about the risks of cancer and the benefits of screening	Group education^a^: webinar format Small media^a^: key takeaways and community resources shared with attendees during and after the event	One-on-one education^a^: clinicians provided education as needed during appointments Small media^a^: brochures and resources provided in post-appointment lounge
Difficulty navigating the healthcare system	Group education^a^: discussion topics included tips for self-advocacy within the healthcare system	Reduce structural barriers^a^: registration support via phone connecting to a dedicated staff member
Language barriers		Reduce structural barriers^a^: on-site language interpreters (2023 only)
Religious beliefs		Reduce structural barriers^a^: female service providers
Transportation difficulties	Reduce client out-of-pocket costs^a^: virtual format to eliminate the need for travel and allow access across the country and beyond	Reduce client out-of-pocket costs^a^: offered transportation support via taxi chits, transit passes, or hospital parking passes
Lack of affordable childcare to attend appointments	Reduce client out-of-pocket costs^a^: virtual event, presented in the evening and recorded to allow later viewing (2022, 2023)	Reduce client out-of-pocket costs^a^: on-site childcare, free of charge, as needed (2023 only)
Other structural barriers and out-of-pocket costs	Reduce structural barriers and out-of-pocket costs^a^: - Free event to maximize access - Events run for 90–120 min on a weekday evening to maximize attendance for participants unavailable during typical business hours	Reduce structural barriers^a^: events run until 6 pm to accommodate a limited number of patients who cannot attend during business hours
Fear of cultural insensitivity or discrimination from providers	Speakers (clinicians and patient volunteers) who are Black and address the experience of discrimination and bias within healthcare	All staff underwent training in cultural safety and anti-oppression before the event Pre- and post-appointment lounges for patients to relax, engage in arts-based activities or mindfulness sessions, and speak informally with staff, clinicians, or community partner representatives Client incentives^b^: - Refreshments and culturally relevant meal - Gift bags
The stigma of cancer within the African diaspora	Digital stories of Black women with lived experience of cancer, produced in partnership with CCS	Creation of a celebratory atmosphere to encourage discussion about cancer and screening Black and other racialized staff to serve as on-site event navigators
Fear of physical pain and discomfort	Speakers (clinicians and patient volunteers) acknowledge and address the fears that often prevent women from seeking screening	Black and other racialized staff to serve as on-site event navigators who can alleviate some pre-appointment fears

^a^Intervention components that have enough supporting evidence to be recommended by the Guide to Community Preventive Services (2023) as effective for increasing breast and/or cervical cancer screening

^b^Intervention components that do not have enough evidence for the Guide to Community Preventive Services (2023) to make a firm conclusion about their effectiveness for increasing breast and/or cervical cancer screening

*BHFBW* Breast/Best Health for Black Women, *CCS* Canadian Cancer Society

Some key elements in the planning timeline for these events included contracting a virtual event support service, confirming speakers and obtaining consent for content release, setting up the registration platform, finalizing the program, creating digital stories, creating communication assets (e.g., flyers—print and digital formats, Zoom background), developing the “run of show” (a detailed, timed list of every element of the program), scheduling email reminders for registrants, scheduling rehearsals with speakers, gathering resources to be shared with attendees, developing the post-event evaluation questionnaire, providing honoraria for speakers, and sharing the event recording. The agenda for both events is outlined in Table 2 with additional details in Online Resource 2: Detailed Program for BHFBW 2023.

**Table 2:** Overview of the agenda for the 2022 and 2023 educational events

Item	Breast Health for Black Women 2022	Best Health for Black Women 2023
Keynote presentation	Empowering Black Women through Knowledge	The Breast Journey of Black Women
Digital patient stories	1. Personality 2. Not if…but When	1. Calculated Risks 2. Rising of Dawn 3. Letting My Spirit Guide Me
Panel discussion	Breast Cancer Prevention, Screening and Early Detection for Black Women: Knowledge as Self-Empowerment	Breast Cancer and Breast Health ▪ Self-examination and screening ▪ Types, stages, signs of illness ▪ Risks and cancer care resources for Black women
Panel discussion	Taking a Closer Look at Genetics and Family History	Gynecological Health ▪ Cancers and fibroids ▪ Self-advocacy
Panel discussion	Self-Advocacy through Uncovered: A Breast Recognition Project	

### Promotion and delivery

We developed communication assets, primarily posters and graphics optimized for various social media platforms, using imagery created to represent the “brand” for this stream of the Centre’s work (see EBC logo and masthead in Online Resource 2). We utilized Eventbrite for registration and promoted the events via social media channels of WCH (paid campaigns) and our community partners (organic posts). Additionally, information was shared through various digital modalities (WCH website, large screen in the WCH atrium, e-newsletters to WCH staff, emails to relevant organizations, community groups, and individuals) and print media (posters displayed around WCH and shared with community partners; postcards distributed by community partners).

In 2022, there were 733 registrants and 454 attendees for the event (62% attendance). In 2023, registrants increased to 1091 and, of those, 459 attended (42%). See Online Resource 1 for more details. In general, respondents to the post-event questionnaire expressed high levels of satisfaction. Table 3 outlines some key challenges and lessons learned from delivering these events.

**Table 3:** Summary of key challenges and lessons learned for the BHFBW events

Project phase	Challenge	Lessons learned and future considerations
Preparation and programming	Producing large-scale virtual events requires expertise	The added cost of a virtual event planning service to assist with execution of these events is worthwhile to ensure that the events run smoothly
	Recruiting and retaining speakers	• Begin at least 3 months before the event to secure availability of busy professionals • Reach out to personal and professional networks with logistical details, event goals, and speaker requirements
	Preparing speakers for the event	• Ensure requirements, specifically any preparatory meetings or rehearsals, are clear to speakers and scheduled as early as possible • Engage panelists in selecting the questions to be asked during the live event
Delivery	Some registrants were unable to connect to the webinar because they could not find the webinar link	• Send a just-in-time reminder email that includes the webinar link • Explore whether using Zoom as the registration platform can streamline the process
	Minor production issues (such as playing videos from YouTube and synchronization issues with slides)	• Use mp4 recordings instead of web links to play videos • Ensure presenters and slides are either both recorded or both live
	Limited time to allow all panelists to share their perspectives on each topic Attendees’ varying perspectives on the scope and depth of information provided	• Allow more time in the program for each panel discussion and audience questions • Consider reducing the number of panelists, topics, or segments to produce a more focused event • Consider developing or promoting other events or platforms/resources that provide more detailed information or cover other topics

## Intervention 2: Breast and Cervical Screening for Black Women

### Design and development

Building on other successful programs for promoting cancer screening among Black women in Ontario (Nnorom et al., 2021), our team utilized a community-based approach to create a safe, inclusive, culturally affirming space for Black women to undergo screening and learn about early detection of breast and cervical cancers. The objective of these events was to provide 25–30 mammograms and 20–25 Pap tests, with appropriate follow-up care, to Black women from the Greater Toronto Area. Online Resource 1 includes the logic model for these events and Table 1 indicates the relationships between selected design elements and barriers to cancer screening. We conceptualized the intervention elements for these events as belonging to one of three categories: patient supports, staff involvement, and curated atmosphere. In creating a curated atmosphere, we leaned on Afro-centric principles (Gebremikael et al., 2022) to infuse joy, dignity, community, and fellowship into the event, most notably through providing refreshments and meals, spaces for lounging, music, event navigators, and accommodation of uninsured patients.

Planning for this event utilized more internal hospital resources to coordinate the relevant clinical departments and staff serving as event navigators. Key elements of planning included working with the diagnostic imaging department to select a date where multiple rooms were available to be booked for event attendees, developing promotional materials, curating a list of community organizations/groups with which to share information, launching promotional activities, monitoring the registration progress, and recruiting and scheduling event navigators.

### Promotion and delivery

We developed “branded” communication assets with link to a registration page and shared these using a tailored approach. We asked our community partners, other community organizations, and professional and personal contacts to share printed postcards as well as digital and printed posters in places where these were most likely to reach the target audience. This included community centres and health fairs serving more racialized neighbourhoods, Facebook groups for Black people, and churches or mosques with predominantly Black or racialized congregants. Additionally, we prepared and supplied a social media toolkit specifically for these groups since they were more likely to reach the target audience.

Once registered, women were triaged by a clinical team and eligible registrants were scheduled for their appointment(s). In the 2 weeks before the event, all patients received 1–2 phone calls or an email to provide necessary information and confirm any required accommodations.

In 2022, 46 of 47 registered patients attended the event, and in 2023, 48 of 56 attended. Data from post-event questionnaires indicated that we successfully created a supportive, culturally affirming atmosphere, and that the experience positively changed attendees’ expectations for healthcare (additional details in Online Resource 1). Table 4 outlines some key challenges and lessons learned.

**Table 4:** Summary of challenges and lessons learned for the Breast and Cervical Cancer Screening for Black Women events

Challenge	Lessons learned and future considerations
Ensuring attendees did not go directly to the clinical areas for their appointments instead of first checking in with event staff	Reminder calls and signage were insufficient. Implement multiple systems, including more direct communication to teams on clinical floors and improved signage to ensure that patients check-in as intended
Minor technological issues with malfunctioning equipment	Expect glitches when setting up new, temporary clinical and clerical spaces. All equipment should be tested in advance
In 2023, there were some unexpected attendees as several women attended with school-age children who remained with them during their appointments	Ask attendees more explicitly, in advance, about the need for accommodation and plan for extra meals and activities
Late/early arrivals by interpreters and/or patients disrupted the planned assignment of interpreters Some interpreters were unaware of the specific requirements of providing interpretation for this event	Interpreters require more details/context about the event and the requirements to ensure a seamless process. Offer an orientation meeting or detailed document to interpreters/their agency in advance of the event Consider: - Increasing the time allotted for these appointments (30 + min) to allow for any unexpected challenges - Hiring a small group of interpreters who are on-call all day to accommodate scheduling changes - Recruiting multilingual staff who may be willing to provide joint navigation/interpretation service

## Discussion

In delivering these events, we considered not only relevant health conditions but also the barriers that patients may encounter when seeking care, information, or support. This holistic approach to healthcare requires acknowledgement that health is not just a matter of medical treatment but an interplay of social, economic, and cultural factors. In fact, a recent review of strategies to address racial and ethnic disparities in health reported that 44% of studies included cultural adaptation and 39% reported community involvement (Lamina et al., 2024). Cultural adaptations included activities such as availability of an interpreter, use of culturally aware peers, and use of community health workers who share sociodemographic characteristics with the patient population. While we leaned on representation and Afro-centric principles to curate a culturally affirming atmosphere for our events, practitioners may consider other approaches. For example, exploring the Picker Principles of Person-Centred Care with particular attention to “Emotional support, empathy and respect” may yield similar results for other groups (Picker Institute, 2024). Another approach is to explore the concept of “welcome” which encompasses a range of practices, both interpersonal and political, that demonstrate a positive engagement with difference and convey appreciation or pleasure at the presence of another (Darling, 2018). Though the concept of “welcome-ability” was developed in relation to communities and the integration of immigrants (Esses et al., 2010; Ravanera et al., 2013), one may consider how an event or institution might improve its welcome-ability. Given that accessible and suitable healthcare is ranked within the top ten characteristics of a community that is welcoming for immigrants and other newcomers (Esses et al., 2010), our provision of preventive health services to people who are uninsured and/or unattached to a primary care provider—via the screening event—may be an example of a program that improves outcomes related to this characteristic of welcoming communities.

Considering the importance of cultural factors for our target population, a key component was our partnership with various community groups. Pre-existing relationships with our community partners allowed us to quickly draw on their support and expertise. However, if those relationships do not yet exist, practitioners must be prepared to dedicate time to their development. Community partners can validate or challenge data that suggest the presence of health disparities, provide context for the mechanisms leading to disparities, and guide practitioners toward solutions that are likely to be impactful and acceptable to the equity-deserving group.

Though it is possible to expend significant cost, time, and resources to design similar events, it is not necessary. If resources are scarce, practitioners can implement individual intervention elements to make small but meaningful impacts for patients. Regardless of the scale of the intervention, it is important to recognize that institutional support (at directorial and managerial levels) is required. We attribute the support we received for these initiatives to the culture of equity that has been cultivated at WCH. For practitioners in organizations without such a culture, we encourage them to start small by looking for alignment between existing strategic priorities and evidence of below-target clinical outcomes for a specific population, and then pursuing an equity-focused quality improvement approach to implementing potential solutions (Green et al., 2010).

Moving forward, we will work towards expanding the audience for BHFBW outside Ontario through connecting with like-minded organizations in other provinces/territories. For the screening event, we will tailor promotional activities to reach specific groups with greater need for accessible health services, e.g., newcomers. We have shared the outcomes of these programs via conference presentations and hope that sharing our reflections here will stimulate consideration of similar approaches by others and adaptation for other underserved populations. We also implore researchers and policy makers to support the expeditious implementation of race-based data collection so that we can more accurately capture what changes, if any, initiatives like these are making to the health behaviours and outcomes of Black women and other equity-deserving groups.

Finally, we encourage practitioners, researchers, and policy makers to begin/continue conversations about the importance of anti-oppression for delivering culturally safe care and achieving health equity (Curtis et al., 2019; MacKenzie & Hatala, 2019). Historically, the healthcare system and its actors have imposed values of the dominant majority on marginalized communities. Past attempts at addressing the ways in which this imposition of values have perpetuated health inequities often lacked a thorough examination of the social determinants of health and underlying power structures that link these determinants to inequities (Curtis et al., 2019). While we offer our initiatives as examples of practices to improve the health of a particular group while working with community organizations, we acknowledge that it is not a substitute for the collective work of adopting an anti-oppressive lens to identify and dismantle oppressive power structures and hold those in power to account. To this end, a next step for us may be to evaluate our relationships with community partners to ensure that they are sustainable and remain mutually beneficial and explore how we can move towards more power-sharing. Making long-term progress on health inequities at a population level will require operationalization of anti-oppression at the individual, institutional, and system levels.

## Implications for policy and practice

What are the innovations in this policy or program?

- We adapted a relatively new practice (i.e., large virtual events) to address some of the barriers that limit Black women’s access to culturally affirming and accessible information and resources about breast and gynecological cancers and other health concerns.
- We present a detailed approach to developing and implementing programs, in partnership with community organizations, that improve access to cancer screening, information, and care for Black women.

What are the burning research questions for this innovation?

- What are the barriers and facilitators for health organizations to engage in activities intended to reduce inequities in health?
- How can initiatives like the one we describe operationalize an anti-oppression lens and enact power-sharing with community members and organizations?
- What is needed to expedite the development of standards for race-based data collection and the implementation of race-based data collection in Ontario and across Canada?
- Will more initiatives like the ones we describe ultimately result in better outcomes for Black women in Canada diagnosed with breast or cervical cancer?

## Supplementary Information

Below is the link to the electronic supplementary material.

Supplementary file1 (PDF 457 KB)

Supplementary file2 (PDF 2416 KB)

## Data Availability

Not applicable.

## Footnotes

1 Co-creators of Every Breast Counts developed a working definition of “Black women” that has been adopted by the Centre for all our subsequent programming. We use the term “Black women” to refer to women of African ancestry or those who self-identify as such, including those who identify as Black, African, Caribbean, African Canadian, melanated, members of the African diaspora, and others.

## References

[CR1] Blackwell, D. L., Martinez, M. E., & Gentleman, J. F. (2008). Women’s compliance with public health guidelines for mammograms and Pap tests in Canada and the United States: An analysis of data from the Joint Canada/United States Survey of Health. *Women’s Health Issues,**18*(2), 85–99. 10.1016/j.whi.2007.10.006 18182305 10.1016/j.whi.2007.10.006

[CR2] Borkhoff, C. M., Saskin, R., Rabeneck, L., Baxter, N. N., Liu, Y., Tinmouth, J., & Paszat, L. F. (2013). Disparities in receipt of screening tests for cancer, diabetes and high cholesterol in Ontario, Canada: A population-based study using area-based methods. *Can J Public Health,**104*(4), e284-290. 10.17269/cjph.104.3699 24044467 10.17269/cjph.104.3699 PMC6973949

[CR3] Canadian Cancer Statistics Advisory Committee in collaboration with the Canadian Cancer Society, Statistics Canada, and the Public Health Agency of Canada. (2021). *Canadian Cancer Statistics 2021*. Retrieved July 31, 2023, from https://cdn.cancer.ca/-/media/files/research/cancer-statistics/2021-statistics/2021-pdf-en-final.pdf

[CR4] Curtis, E., Jones, R., Tipene-Leach, D., Walker, C., Loring, B., Paine, S. J., & Reid, P. (2019). Why cultural safety rather than cultural competency is required to achieve health equity: A literature review and recommended definition. *International Journal for Equity in Health,**18*(1), 174. 10.1186/s12939-019-1082-3 31727076 10.1186/s12939-019-1082-3 PMC6857221

[CR5] Darling, J. (2018). The fragility of welcome – Commentary to Gill. *Fennia - International Journal of Geography,**196*(2), 220–224. 10.11143/fennia.75756

[CR6] Esses, V. M., Hamilton, L. K., Bennett-AbuAyyash, C., & Burstein, M. (2010). *Characteristics of a welcoming community* (report prepared for the Integration Branch of Citizenship and Immigration Canada). Retrieved July 9, 2024, from http://p2pcanada.ca/wp-content/uploads/2011/09/Characteristics-of-a-Welcoming-Community-11.pdf

[CR7] Evans, J., Butler, L., Etowa, J., Crawley, I., Rayson, D., & Bell, D. G. (2005). Gendered and cultured relations: Exploring African Nova Scotians’ perceptions and experiences of breast and prostate cancer. *Research and Theory for Nursing Practice,**19*(3), 257–273. 10.1891/rtnp.2005.19.3.257 16144243 10.1891/rtnp.2005.19.3.257

[CR8] Gebremikael, L., Sicchia, S., Demi, S., & Rhoden, J. (2022). Afrocentric approaches to disrupting anti-Black racism in health care and promoting Black health in Canada. *CMAJ,**194*(42), E1448–E1450. 10.1503/cmaj.220456 36316022 10.1503/cmaj.220456 PMC9828881

[CR9] Giaquinto, A. N., Miller, K. D., Tossas, K. Y., Winn, R. A., Jemal, A., & Siegel, R. L. (2022). Cancer statistics for African American/Black people 2022. *CA: A Cancer Journal for Clinicians,**72*(3), 202–229. 10.3322/caac.21718 35143040 10.3322/caac.21718

[CR10] Green, A. R., Tan-McGrory, A., Cervantes, M. C., & Betancourt, J. R. (2010). Leveraging quality improvement to achieve equity in health care. *Joint Commission Journal on Quality and Patient Safety,**36*(10), 435–442. 10.1016/s1553-7250(10)36065-x 21548504 10.1016/s1553-7250(10)36065-x

[CR11] Guide to Community Preventive Services. (2023). *CPSTF findings for cancer prevention and control*. Retrieved April 26, 2024, from https://www.thecommunityguide.org/pages/task-force-findings-cancer-prevention-and-control.html

[CR12] Hashi, A., Khan, R., Appiahene-Afriyie, A., Barker, D., Higgins, T., Khalil, I., Pottinger, D., Spencer, S., Springer, L., Covelli, A., Goulbourne, E., Heisey, R., Wu, M., & Lofters, A. (2023). Enhancing the care experiences of Black women along the breast cancer journey: Meaningfully engaging breast cancer survivors to co-create a targeted, culturally relevant resource hub. *University of Toronto Journal of Public Health*,* 4*(2). 10.33137/utjph.v4i2.39024

[CR13] Health Quality Ontario. (2018). *Measuring Up 2018*. Retrieved July 8, 2024, from http://www.hqontario.ca/Portals/0/Documents/pr/measuring-up-2018-en.pdf

[CR14] Hwee, J., & Bougie, E. (2021). Do cancer incidence and mortality rates differ among ethnicities in Canada? *Health Rep,**32*(8), 3–17. 10.25318/82-003-x202100800001-eng 34405970 10.25318/82-003-x202100800001-eng

[CR15] Lamina, T., Abdi, H. I., Behrens, K., Call, K., Claussen, A. M., Dill, J., Grande, S. W., Houghtaling, L., Jones-Webb, R., Nkimbeng, M., Parikh, R., Rogers, E., Sultan, S., Widome, R., Wilt, T. J., Butler, M. (2024). *Strategies to address racial and ethnic disparities in health and healthcare: An evidence map* (AHRQ Publication No. 24-EHC017). Agency for Healthcare Research and Quality. 38687837

[CR16] Lofters, A., Guilcher, S., Glazier, R. H., Jaglal, S., Voth, J., & Bayoumi, A. M. (2014). Screening for cervical cancer in women with disability and multimorbidity: A retrospective cohort study in Ontario, Canada. *CMAJ Open,**2*(4), E240-247. 10.9778/cmajo.20140003 25485249 10.9778/cmajo.20140003 PMC4251502

[CR17] Lofters, A. K., Mark, A., Taljaard, M., Green, M. E., Glazier, R. H., & Dahrouge, S. (2018). Cancer screening inequities in a time of primary care reform: A population-based longitudinal study in Ontario, Canada. *BMC Family Practice,**19*(1), 147. 10.1186/s12875-018-0827-1 30157772 10.1186/s12875-018-0827-1 PMC6116433

[CR18] Lofters, A. K., Kopp, A., Vahabi, M., & Glazier, R. H. (2019). Understanding those overdue for cancer screening by five years or more: A retrospective cohort study in Ontario, Canada. *Preventive Medicine,**129*, 105816. 10.1016/j.ypmed.2019.105816 31445111 10.1016/j.ypmed.2019.105816

[CR19] Louwman, W. J., van de Poll-Franse, L. V., Fracheboud, J., Roukema, J. A., & Coebergh, J. W. (2007). Impact of a programme of mass mammography screening for breast cancer on socio-economic variation in survival: A population-based study. *Breast Cancer Research and Treatment,**105*(3), 369–375. 10.1007/s10549-006-9464-9 17211536 10.1007/s10549-006-9464-9 PMC2190785

[CR20] MacKenzie, L., & Hatala, A. (2019). Addressing culture within healthcare settings: The limits of cultural competence and the power of humility. *Canadian Medical Education Journal,**10*(1), e124–e127. 30949267 PMC6445323

[CR21] Nnorom, O., Sappong-Kumankumah, A., Olaiya, O. R., Burnett, M., Akor, N., Shi, N., Wright, P., Gebreyesus, A., Gebremikael, L., & Lofters, A. (2021). Afrocentric screening program for breast, colorectal, and cervical cancer among immigrant patients in Ontario. *Canadian Family Physician,**67*(11), 843–849. 34772714 10.46747/cfp.6711843 PMC8589122

[CR22] Ontario Agency for Health Protection and Promotion (Public Health Ontario), Abdi, S., & Mensah, G. (2016). *Focus on: Logic model–A planning and evaluation tool*. Queen’s Printer for Ontario. Retrieved August 17, 2023, from https://www.publichealthontario.ca/-/media/documents/f/2016/focus-on-logic-model.pdf

[CR23] Picker Institute. (2024). *The Picker Principles of Person Centred Care*. Picker Institute. Retrieved June 11, 2024 from https://picker.org/who-we-are/the-picker-principles-of-person-centred-care/

[CR24] Public Health Agency of Canada. (2020). *Social determinants and inequities in health for Black Canadians: A snapshot 2020* (978–0–660–35783–6).

[CR25] Ravanera, Z. R., Esses, V., & Fernando, R. (2013). Integration and “welcome-ability” indexes: Measures of community capacity to integrate immigrants. *Discussion Paper Series/Documents de travail. Population Change and Lifecourse Strategic Knowledge Cluster/Un Réseau stratégique de connaissances : Changement de population et parcours de vie.** 1*(1), Article 6. Retrieved July 9, 2024, from https://ir.lib.uwo.ca/pclc/vol1/iss1/6

[CR26] Statistics Canada. (2022). *Canada at a glance, 2022* (2022001). Retrieved June 1, 2024, from https://www150.statcan.gc.ca/n1/en/catalogue/12-581-X2022001

[CR27] Tjepkema, M., Christidis, T., Olaniyan, T., & Hwee, J. (2023). Mortality inequalities of Black adults in Canada. *Health Rep,**34*(2), 3–16. 10.25318/82-003-x202300200001-eng 36791269 10.25318/82-003-x202300200001-eng

