# Supplementary material for: Initiatives to increase breast and cervical cancer–related knowledge, screening, and health behaviours among Black women
Source: Can J Public Health. 2024 Oct 22;116(1):100–8. doi: 10.17269/s41997-024-00953-y (PMC11868473; doi:10.17269/s41997-024-00953-y)
Supplement: Supplementary file 1 — Supplementary file1 (PDF 457 KB) [file 41997_2024_953_MOESM1_ESM.pdf]

ONLINE RESOURCE 1

Program Evaluation of Virtual Educational and Screening Events for Black Women

Article Title: Initiatives to increase breast and cervical cancer-related knowledge, screening, and health behaviours among Black women

Journal Name: Canadian Journal of Public Health – Innovations in Policy and Practice section

Methods

Evaluative Approach

For both interventions, we focused on conducting an *impact evaluation* which "measures the immediate effect of the program, i.e. whether it meets its objectives, by assessing what changes, if any, have occurred in the predisposing, reinforcing, and enabling factors, targeted behaviours and the environments" (O'Connor-Fleming et al., 2006). Our impact evaluations were informed by *Outputs* and *Short-term outcomes* in the logic models outlined in Appendices 1 and 2 (Ontario Agency for Health Protection and Promotion (Public Health Ontario) et al., 2016), as well as the RE-AIM framework, particularly *Reach* and *Effectiveness*. *Reach* is helpful to explore whose health, health behaviours, health-related knowledge, and/or intentions we hope to change via the intervention, who is exposed to the intervention and who actually participates in the intervention (Glasgow & Estabrooks, 2018). *Effectiveness* is helpful to outline what impacts the intervention had on important outcomes and what aspects of the intervention were necessary to achieve those impacts (Glasgow & Estabrooks, 2018). In Table 1, we outline the measures evaluated from the logic models and RE-AIM after accounting for practical considerations.

Data Collection and Analysis

Breast/Best Health for Black Women

Demographic information was collected at the time of online registration via Eventbrite. Data on attendance and engagement were collected from Zoom – the platform used to host the events. Other relevant data (e.g., satisfaction, feedback) were collected from a post-event questionnaire hosted on Survey Monkey®. Descriptive statistics were used to summarize responses to multiple choice questions and text from open-ended questions were reviewed for recurring ideas and impactful quotes.

Breast and Cervical Cancer Screening for Black Women

A post-event pen-and-paper questionnaire was administered after attendees completed their appointments that included questions about women's experience of the event, the accommodations provided, how the experience made them feel and whether it might change their future behaviour. Descriptive statistics were used to summarize the results. We also utilized clerical and clinical records to extract metrics about participation and follow-up care. Only aggregated data about program participation and clinical care are reported. Text from open-ended questions were reviewed for recurring themes or ideas, and impactful quotes.

Table 1. Evaluative measures informed by project logic models and a pragmatic application of RE-AIM

Evaluative Component	Breast/Best Health for Black Women	Breast and Cervical Cancer Screening for Black Women
**WHO?**	Number of registrants and attendees	Number of registrants and attendees
	Ways in which registrants heard about the events	Ways in which registrants heard about the events
	Demographic characteristics of registrants (e.g., ethnicity, race, age, geographic region)	Demographic characteristics of attendees (e.g., ethnicity, race, age, geographic region)
**WHAT?**	Number of guests (patients, healthcare professionals and community partners) who delivered in the program	Number of women who received cancer screening via mammogram, Pap test, and breast ultrasound.
	Average time that attendees tuned in	Number of women who received follow-up care
		Number of women who received transportation support
		Number of women who utilized childcare
		Number of women who utilized an interpreter
**IMPACT**	Creating a safe space to have questions answered	Creating a safe space to undergo cancer screening
	Attendees feeling supported with regards to their health	Attendees feeling supported with regards to their health
	Attendees having a better understanding of resources that can be accessed	Attendees feeling empowered by the experience
	Attendees being able to identify actions that can be beneficial to their health	Creating opportunities for attendees to have all their questions answered
	Attendees' knowledge about screening, risk factors, and symptoms for breast and gynecological cancers and conditions	Decreasing stress associated with hospitals and cancer screening
	Attendees' understanding of when to seek mental health support for health challenges	Using navigators to foster a welcoming atmosphere at the hospital
	Attendees' understanding of how to find culturally affirming healthcare providers	Attendees' likelihood of being screened when next due
	Number of attendees motivated to make changes in their life	Attendees' satisfaction with language support

Outcomes

Breast/Best Health for Black Women

Who. In 2022, there were 733 registrants and 454 attendees for the event (62% attendance). In 2023, the number who registered increased to 1091 and, of those, 459 attendees (42% attendance). Characteristics of the events' registrants are outlined in Table 2. Approximately one fifth of attendees in each year identified as healthcare providers. In both years, the top two ways that registrants heard about the event were through media/social media (33% in 2022; 46% in 2023) and from a friend/colleague/family member (32%; 25%).

Table 2. Characteristics of registrants for the virtual educational events in 2022 and 2023

Characteristics of Registrants		2022 (N = 733)	2023 (N = 1091)
**Gender identity, n (%)**			
	Female (cis woman)	694 (94.7%)	1012 (92.8%)
	Male (cis man)	12 (1.6%)	16 (1.5%)
	Trans	0 (0.0%)	0
	Genderfluid, genderqueer or gender nonbinary	5 (0.7%)	6 (0.5%)
	Two-spirit (Indigenous)	0 (0.0%)	0
	Another gender identity	0 (0.0%)	1 (0.1%)
	Unsure/Do not know	1 (0.1%)	1 (0.1%)
	Prefer not to answer	20 (2.7%)	55 (5.0%)
**Ethno-racial background, n (%)**			
	African-Canadian	143 (19.5%)	215 (19.7%)
	Afro-Caribbean	278 (37.9%)	418 (38.3%)
	North African	9 (1.2%)	4 (0.4%)
	Central African	4 (0.5%)	5 (0.5%)
	South African	6 (0.8%)	13 (1.2%)
	East African	17 (2.3%)	30 (2.7%)
	West African	28 (3.8%)	71 (6.5%)
	First Nations / Inuit / Métis / Indigenous	1 (0.1%)	1 (0.1%)
	Multiple ethno-racial backgrounds	81 (11.1%)	148 (13.6%)
	Other	110 (15.0%)	108 (9.9%)
	Prefer not to answer	56 (7.6%)	78 (7.1%)
**Age, n (%)**			
	Under 18	7 (1.0%)	5 (0.4%)
	19-25	55 (7.5%)	69 (6.3%)
	26-30	71 (9.7%)	108 (10.0%)
	31-35	84 (11.5%)	130 (12.0%)
	36-40	95 (13.0%)	169 (15.5%)
	41-45	140 (19.1%)	177 (16.2%)
	46-50	97 (13.2%)	168 (15.4%)
	51-55	91 (12.4%)	145 (13.3%)
	56+	93 (12.7%)	120 (11.0%)
**Geographic region, n (%)**			
	Ontario	593 (80.9%)	826 (75.7%)
	Another Canadian province	80 (10.9%)	206 (18.9%)
	Outside Canada	60 (8.2%)	59 (5.4%)

What. Attendees tuned in for an average of 62 min in 2022 and 80 min in 2023. Both these events were delivered with the support of volunteers who were patients and healthcare professionals as well as community partners – 14 in 2022 and 18 in 2023. These volunteers shared their personal and lived experiences of navigating the healthcare system or supporting someone else to navigate the systems or their research-based knowledge and professional experiences of breast and gynecological health.

Table 3. Selected results from the post-event questionnaires indicating impact of the educational interventions. Percentages were calculated using the total responses for each item as the denominator. Cells marked as "NA" (not applicable) were not asked on the questionnaire for that year.

Statement on post-event questionnaire	Agreed or strongly agreed, n (%)		Not sure, n (%)		Disagreed or strongly disagreed, n (%)		Not Applicable, n (%)	
	2022	2023	2022	2023	2022	2023	2022	2023
The event created a safe space to have my questions answered.	55 (90.2%)	73 (88.0%)	0 (0%)	4 (4.8%)	0 (0%)	4 (4.8%)	6 (9.8%)	2 (2.4%)
Seeing an event specifically for Black women made me feel supported on my health.	56 (87.5%)	78 (92.9%)	1 (1.6%)	2 (2.4%)	0 (0%)	1 (1.2%)	7 (10.9%)	3 (3.6%)
I have a better understanding of resources that exist that I can access on my health journey.	55 (88.7%)	77 (91.7%)	2 (3.2%)	3 (3.6%)	0 (0%)	2 (2.4%)	5 (8.1%)	2 (2.4%)
The event helped me identify actions that could be beneficial to my health.	54 (93.1%)	80 (95.2%)	0 (0%)	1 (1.2%)	0 (0%)	2 (2.4%)	4 (6.9%)	1 (1.2%)
I feel more informed about the importance of early screening and early detection for breast cancer.	85 (93.4%)	73 (93.6%)	1 (1.1%)	2 (2.6%)	0 (0%)	1 (1.3%)	5 (5.5%	2 (2.6%)
I have a better understanding of the role of family history and genetics in my health.	85 (93.4%)	NA	0 (0%)	NA	0 (0%)	NA	6 (6.6%)	NA
I have a better understanding of the role of family history and other risk factors in breast health.	NA	73 (94.8%)	NA	1 (1.3%)	NA	2 (2.6%)	NA	1 (1.3%)
I feel more informed about the signs and symptoms of uterine cancer and when to request screening.	NA	64 (83.1%)	NA	5 (6.5%)	NA	2 (2.6%)	NA	6 (77.9%)
I feel more informed about fibroids, including risk factors, when to be concerned, and treatment options.	NA	60 (79.0%)	NA	6 (7.9%)	NA	3 (4.0%)	NA	7 (9.2%)
I feel more informed about the signs and symptoms of endometriosis.	NA	59 (77.6%)	NA	8 (10.5%)	NA	2 (2.6%)	NA	7 (9.2%)
I have a better understanding of when to seek mental health support for health challenges I may face/have faced and how to find that support.	NA	68 (88.3%)	NA	2 (2.6%)	NA	3 (3.9%)	NA	4 (5.2%)

Impact. In both years, approximately one fifth of attendees (92 and 89, respectively) responded to our post-event evaluation questionnaire. In general, attendees expressed a high level of satisfaction with the events (Table 3). They appreciated that the events provided some of the knowledge and tools necessary for patients to advocate for equity in their own care. For example, a 2023 attendee noted that the event allowed her "To feel more confident in speaking up for myself with my doctor". Additionally, it prompted professionals/health system leaders to think about how to address inequities in the systemic delivery and practice of cancer care. A 2022 attendee noted that "Being a mammographer. It was an eye opener about disparities that Black women deal with when trying to access breast screening and treatment".

Breast and Cervical Cancer Screening for Black Women

Who. In 2022, 46 of 47 registered patients attended the event (average age: 44.5yr) and 7 patients scheduled their appointments for another day. One unregistered patient also attended and was accommodated. Forty-one attendees (87%) completed the post-event survey. These respondents all identified as cisgender female and 89% of them identified as Black (Figure 1). When asked how they heard about the event, 49% of respondents selected our community partner Women's Health in Women's Hands Community Health Centre (CHC), 30% selected a personal contact and 12% selected a contact at Women's College Hospital.

In 2023, of the 56 registered patients (average age: 41.1yr), 48 patients attended. Of the 8 no-shows and cancellations, 3 completed their appointments later. Forty attendees (83%) completed the post-event survey. All but one (98%) identified as cisgender female and, interestingly, only 42% identified as Black (Figure 1). When asked how they heard about the event, Women's Health in Women's Hands CHC (23%) and personal contacts (20%) were again significant sources, but social media emerged as the primary source (26%).

What. Figure 2 shows the number of screening tests provided at each event. In 2022, 30 participants (64%) received transportation support in the form of transit tokens, taxi chits or a parking voucher while only ten participants (20%) received transportation support in 2023. We engaged 5 Spanish-speaking interpreters to support 10 patients in-person at the 2023 event and 1 participant, who self-identified as deaf, requested and received clear masks to allow speech reading. One participant utilized the hospital's onsite childcare program during their appointment.

In keeping with the program's goals, we also provided any necessary follow-up care to women. In 2022, 10 women were called back for additional imaging after their screening mammogram, 5 women were referred for genetic counselling, and 10 were connected to a primary care provider. In 2023, follow-up care included 14 referrals for additional imaging following a screening mammogram, 4 referrals for genetic testing, treating 2 women for an infection, and 2 referrals for human papillomavirus testing.

Impact. Key outcomes from the post-event questionnaire highlighting the impact of these events are described in Table 4 and Figure 3. We also provided space for additional, open-ended feedback on the event. When asked what made the event a success, some participants noted elements of the event's program, accommodations provided and communication from the clinical and planning teams:

- "Emails, [telephone] calls and grab bag made me feel committed to showing up." *(2022 attendee)*
- "Reminder [tele]phone calls, detailed email information, friendly and informative staff and transportation support." *(2022 attendee)*

- “Overall, ongoing communication and reminders. I appreciated that efforts were made to ensure this was an accessible experience” *(2023 attendee)*
- “Support given to Hispanic people.” *(2023 attendee)*

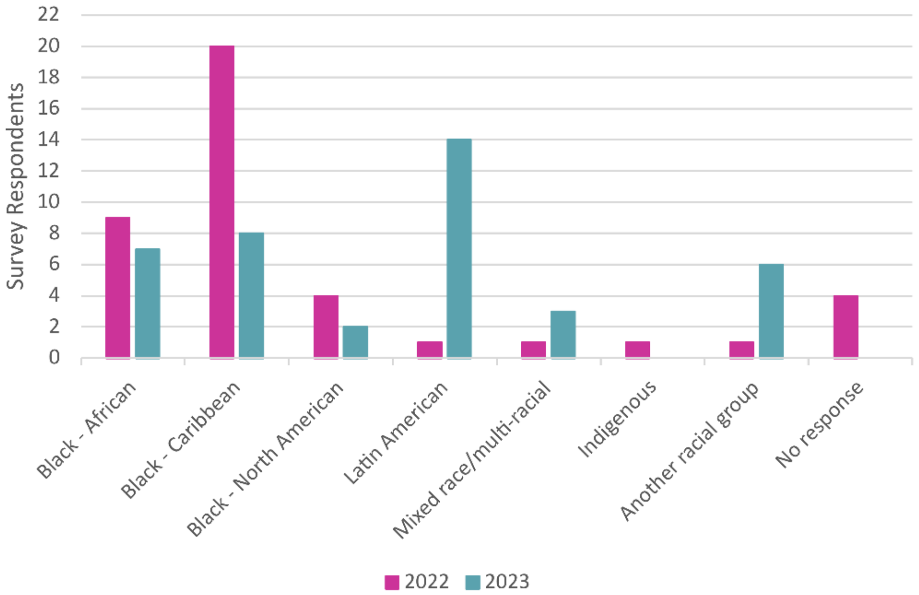

Figure 1. Ethno-racial distribution of survey respondents for both cancer screening events. Note: The “Another racial group” category includes those who selected Middle Eastern, Arab or West Asian, East Asian, South Asian, Southeast Asian, White, Other or Do Not Know. The “No response” category includes those who selected Prefer Not to Answer or left the question blank.

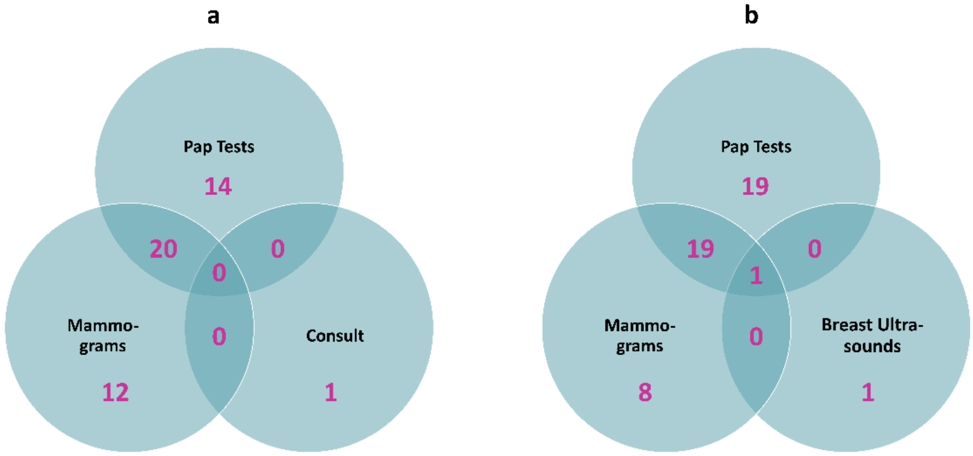

Figure 2. Venn diagrams showing the number and types of screening tests received at the 2022 (a) and 2023 (b) screening events

Table 4. Selected results from the screening event questionnaires indicating impact of the interventions. Percentages were calculated using the total responses for each item as the denominator.

Statement on post-event questionnaire	Agreed or strongly agreed, n (%)		Disagreed or strongly disagreed, n (%)		Not Applicable, n (%)	
	2022	2023	2022	2023	2022	2023
The event created a safe space and environment for me to undergo cancer screening.	41 (100%)	37 (92.5%)	0 (0%)	3 (7.5%)	0 (0%)	0 (0%)
Seeing an event specifically for Black women made me feel supported and heard on my health journey (2022)/with regards to my health concerns (2023).	40 (97.6%)	37 (92.5%)	1 (2.4%)	3 (7.5%)	0 (0%)	0 (0%)
I felt empowered by this experience.	39 (97.5%)	37 (92.5%)	1 (2.5%)	3 (7.5%)	0 (0%)	0 (0%)
The program (mindfulness sessions, arts-based activities, refreshments etc.) decreased the stress associated with hospitals and cancer screening.	36 (87.8%)	37 (92.5%)	0 (0%)	3 (7.5%)	5 (12.2%)	0 (0%)
There were opportunities to have all my questions answered.	40 (97.6%)	37 (92.5%)	0 (0%)	3 (7.5%)	1 (2.4%)	0 (0%)
The use of navigators made me feel welcome at the hospital.	41 (100%)	37 (92.5%)	0 (0%)	3 (7.5%)	0 (0%)	0 (0%)

Some patients also expressed that the creation of cultural safety and the special consideration given to Black women were integral to the event's success:

- "The tailored experience, the 'tender' care.... this setting was very comfortable as well as the warm and welcoming staff." *(2022 attendee)*
- "It was great to see so many Black women in healthcare (although every woman was fantastic!). It felt very welcome here. Thank you!" *(2022 attendee)*
- "Very friendly staff, warm & welcoming. Considered many of the little anxieties that black/BIPOC women might feel, and they made sure to address it." *(2023 attendee)*

Some participants noted that the experience has changed their impression of and/or expectations for healthcare experiences:

- "Because of this experience, my outlook and anxiety about the clinical experience aspect of it has changed!" *(2022 attendee)*
- "Job well done, and I hope to see more events like this for women of colour in the future." *(2022 attendee)*
- "Thank you for existing and for creating this initiative. It's a VIP treatment I've never received." *(2023 attendee)*

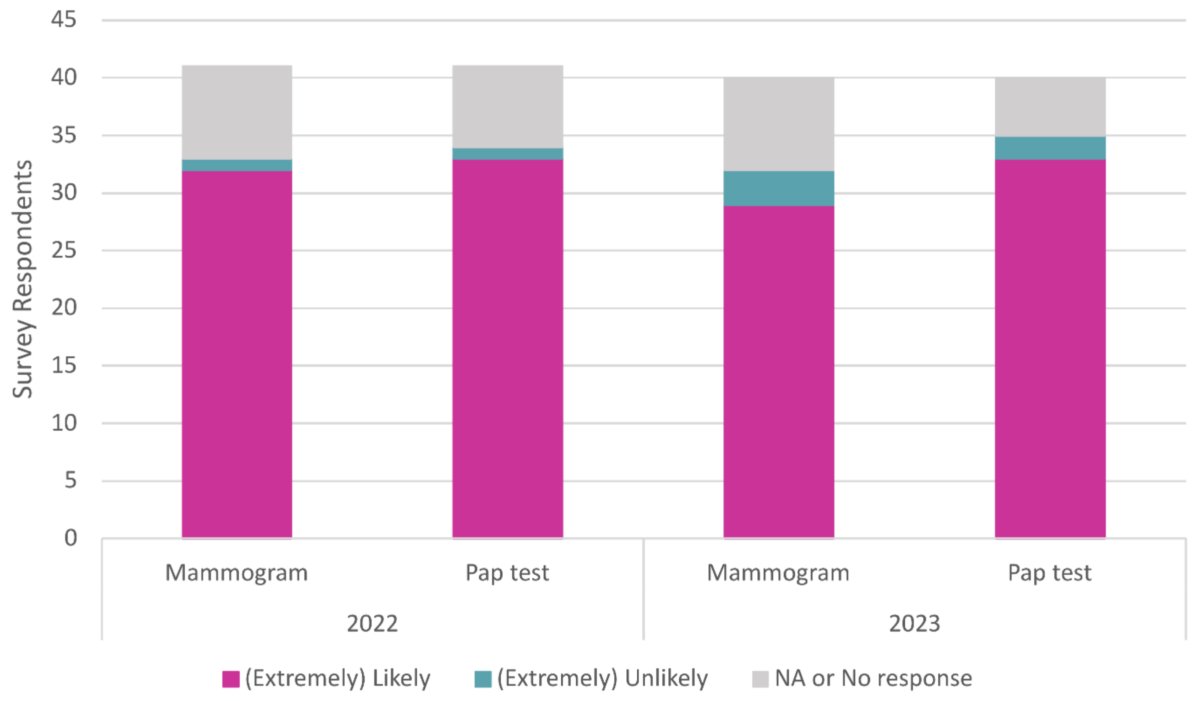

Figure 3. Distribution of responses to the following statements on the screening events' post-event questionnaire: "Based on your experience today, what is the likelihood that you will have a mammogram (or: Pap test) when you are next due for one?". NA: Not applicable

References

Glasgow, R. E., & Estabrooks, P. E. (2018). Pragmatic Applications of RE-AIM for Health Care Initiatives in Community and Clinical Settings. *Preventing Chronic Disease*, *15*. https://doi.org/10.5888/pcd15.170271

O'Connor-Fleming, M. L., Parker, E., Higgins, H., & Gould, T. (2006). A framework for evaluating health promotion programs. *Health Promot J Austr*, *17*(1), 61-66. https://doi.org/10.1071/he06061

Ontario Agency for Health Protection and Promotion (Public Health Ontario), Abdi, S., & Mensah, G. (2016). *Focus On: Logic model–A planning and evaluation tool*. Queen's Printer for Ontario. https://www.publichealthontario.ca/-/media/documents/f/2016/focus-on-logic-model.pdf

Steering Committee for National Working Group on RE-AIM Planning and Evaluation Framework. *What is RE-AIM?* Retrieved 2023 August 18 from https://re-aim.org/learn/what-is-re-aim/

Appendices

Appendix 1. Logic model for the virtual educational events: Breast/Best Health for Black Women

INPUTS	ACTIVITIES	AUDIENCE	OUTPUTS	SHORT-TERM OUTCOMES
• Funding<br>• Women's College Hospital (WCH) staff and volunteers<br>• Staff and volunteers from community partners<br>• Patients and healthcare professionals from the Black community<br>• Technical support to support a large-scale virtual event	• A virtual educational event<br>• Tailored outreach and promotion via community partners<br>• Promotion via on site posters, traditional news outlets, social media, local event listings, and WCH newsletters<br>• A keynote presentation from a Black clinician<br>• Digital stories from Black patients who have experienced breast and/or gynecological cancer<br>• Two moderated panel discussions involving Black clinicians, patient advocates, and/or community partners<br>• Resources from each community partner	• Black women from across Canada, their family, friends and caregivers who are interested in maintaining or concerned about breast health, gynecological health and related cancers or conditions<br>• Healthcare professionals who care for Black women	• # of registrants and attendees of the virtual event<br>• # of patients, healthcare professionals and community partners who delivered keynote presentations, participated in panel discussions, or created a digital story to be shared	• Creating a culturally safe space to have questions answered<br>• Feeling supported and heard with regards to their health concerns.<br>• Understanding of resources available<br>• Identification of actions that can benefit health<br>• Knowledge about screening, risk factors, and symptoms for breast and gynecological cancer and conditions<br>• Understanding when to seek mental health support for health challenges<br>• Understanding how to find culturally affirming healthcare providers<br>• # of attendees motivated to make changes

Appendix 2. Logic model for the Breast and Cervical Cancer Screening for Black Women events

INPUTS	ACTIVITIES	AUDIENCE	OUTPUTS	SHORT-TERM OUTCOMES
• Funding<br>• Staff and volunteers (WCH, community partners)<br>• Time in clinic schedules to accommodate event-related appointments on the same day<br>• Clinical supplies and equipment<br>• Cultural safety/anti-oppression training for staff	• Tailored outreach and promotion via community partners<br>• Promotion via on-site posters, social media, and WCH newsletters<br>• Breast cancer screening via mammography or ultrasound and cervical cancer screening via Pap test<br>• Navigation support from WCH staff<br>• Accommodation for undocumented or uninsured patients<br>• Refreshments and a culturally relevant meal<br>• Arts-based activities and gift bags<br>• On-site social work, clinical support, and resources about breast health<br>• On-site childcare<br>• Transportation support<br>• In-person language interpreters	• Black women in the Greater Toronto Area, who are under- or never-screened for breast and/or cervical cancer	• # women getting a mammogram<br>• # women getting a Pap test<br>• # women getting a breast ultrasound<br>• # women receiving relevant follow-up care (e.g., referrals to genetics)<br>• # women receiving transportation support<br>• # women utilizing childcare<br>• # women utilizing an interpreter and being satisfied	• Creating a culturally safe space to undergo cancer screening<br>• Feeling supported and heard with regards to their health concerns.<br>• Feeling empowered by the experience.<br>• Likelihood of being screened when next due
