# Supplementary material for: Initiatives to increase breast and cervical cancer–related knowledge, screening, and health behaviours among Black women
Source: Can J Public Health. 2024 Oct 22;116(1):100–8. doi: 10.17269/s41997-024-00953-y (PMC11868473; doi:10.17269/s41997-024-00953-y)
Supplement: Supplementary file 2 — Supplementary file2 (PDF 2416 KB) [file 41997_2024_953_MOESM2_ESM.pdf]

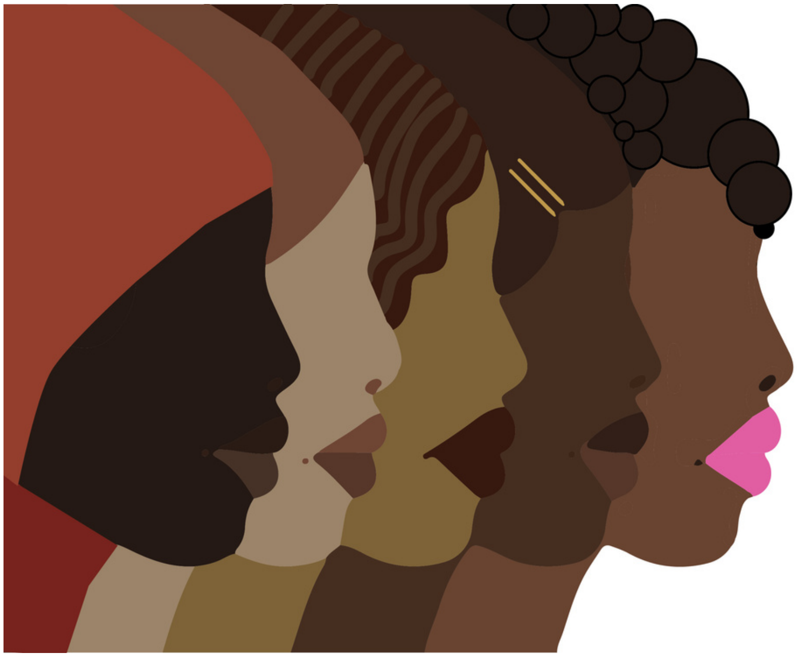

BEST HEALTH FOR BLACK WOMEN

A Conversation about Breast Cancer and Gynecological Health

PRESENTED BY:

THE PETER GILGAN CENTRE FOR WOMEN'S CANCERS

The Olive Branch of Hope Cancer Support Services

rethink BREAST CANCER

WELCOME

WELCOME FROM THE PETER GILGAN CENTRE AND WOMEN'S COLLEGE HOSPITAL

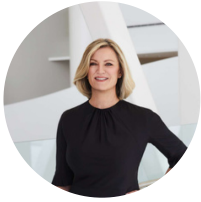

Heather McPherson, President / Corporate Executive Officer (CEO) Women's College Hospital

Heather McPherson is a highly recognized healthcare executive with extensive experience in clinical, academic and health system leadership. Heather is an innovative leader who has been instrumental in establishing clinical programs and services that close the health gaps for patients – at Women's College Hospital and throughout the province. She is a passionate advocate for an equitable, multi-faceted and comprehensive approach to health.

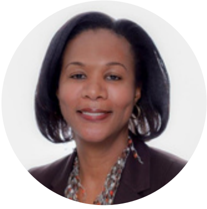

Elaine Goulbourne, Clinical Director, Primary Care & Peter Gilgan Centre for Women's Cancers / Director, Clinical Resources and Performance

Elaine has a Master in health studies from Athabasca University and a Bachelor of Science in Nursing from Laurentian University. She holds an adjunct lecturer appointment with the Lawrence S. Bloomberg Faculty of Nursing at the University of Toronto. Elaine has more than 25 years of healthcare leadership and clinical expertise in the areas of surgery, primary care and women's and children's health.

WELCOME FROM THE OLIVE BRANCH OF HOPE

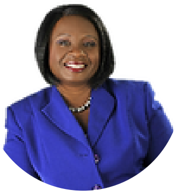

Leila Springer, Founder / Executive Director

Leila Springer is an author, life coach, inspirational and motivational speaker, breast cancer survivor and community activist. She is a Co-Founder of The Olive Branch of Hope (TOBOH), where she volunteers as Executive Director. Living strong and giving back to the community is Leila's motto.

WELCOME FROM RETHINK BREAST CANCER

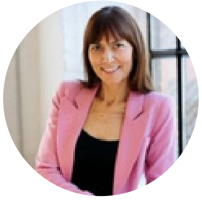

MJ DeCoteau, Founder / Executive Director

MJ DeCoteau is the Founder and Executive Director of Rethink Breast Cancer, the change-maker organization that for 20 years has been inspiring, educating and advocating for people concerned about and affected by breast cancer, helping them live longer and live better.

WELCOME FROM THE CANADIAN CANCER SOCIETY (CCS)

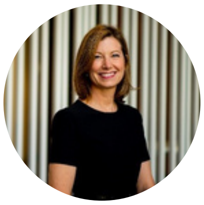

Andrea Seale, Chief Executive Officer

Andrea Seale is the CEO of the Canadian Cancer Society. With more than 20 years of non-profit leadership experience, Andrea is dedicated to empowering people to build the causes that make our world more compassionate, healthy and just.

MAIN SESSION

THE BREAST JOURNEY OF BLACK WOMEN

Dr. Aisha Lofters will present *The Breast Journey of Black Women*, a keynote presentation that will provide information about breast health and share actions that we are all able to take in our day-to-day lives to take care of our well-being.

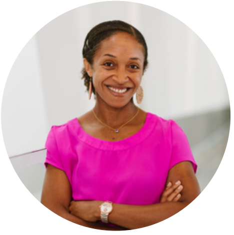

Keynote speaker: Dr. Aisha Lofters
Family Physician at Women's College Hospital Family Practice Health Centre, Medical Director and Chair in Implementation Science Peter Gilgan Centre for Women's Cancers at Women's College Hospital; Associate Professor and Clinician Scientist Department of Family and Community Medicine, University of Toronto

STORIES

The main session will also provide an opportunity to hear stories from survivors as they share their personal health journeys.

CALCULATED RISKS
By Nadia

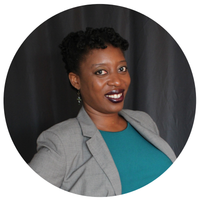

Nadia Headley brings us into her personal journey with breast cancer. She shares a story of strength and resilience; having positive outlook and determination regardless of what cancer would bring. Nadia holds many titles but the most important are Mom and Wife. Nadia is grateful for the willingness and opportunity to share her story so that other young women of African descent understand that it is OK to prioritize their health.

Rising of Dawn
By Dawn

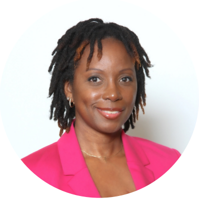

Dawn Barker-Pierre was born in Barbados and immigrated to Canada at a very young age. She is a wife, proud mother of three and a triple negative breast cancer thrive. She has volunteered with The Olive Branch of Hope to encourage, uplift and inspire women. Some of Dawn's most recent accomplishments include Co-creating the Women's College Hospital "Every Breast Counts" webpage, long-standing advocate for earlier screening in the Toronto Star, CTV News Toronto, CityNews and the February 2023 edition of Best Health Magazine. Dawn also serves on the Board of Directors for Circle of Love Community Outreach, a Ministry that feed and clothe the homeless. Being a difference maker is who Dawn is.

Letting my Spirit Guide Me
By Janelle

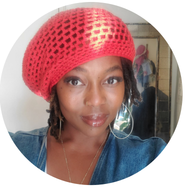

A registered nurse who has worked in obstetrics for 13 years at Mount Sinai hospital. She has endeavoured to expand her nursing skill set by joining the Sexual Assault and Domestic Violence Care Centre. There she has learned the unique ways of nursing through a trauma-informed care perspective and believes that this model should be the standard throughout healthcare. In her spare time she loves to catch up with herself by journaling, bike riding to the lake and therapy sessions. She's recently returned to her love for knitting and discovered her joy for crocheting. Janelle is supported and loved by a tremendous group of people that consistently show up her. For this, she is ultimately thankful.

BEST HEALTH FOR BLACK WOMEN 2023

PANEL 1

BREAST CANCER AND BREAST HEALTH

Moderated by:

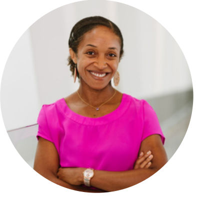

Dr. Aisha Lofters
Family Physician at Women's College Hospital Family Practice Health Centre, Medical Director and Chair in Implementation Science Peter Gilgan Centre for Women's Cancers at Women's College Hospital; Associate Professor and Clinician Scientist Department of Family and Community Medicine, University of Toronto

Aisha is a scientist at the Women's College Research Institute (WCRI), adjunct senior scientist at IC/ES, and an Associate Professor in the Department of Family and Community Medicine at the University of Toronto. She previously held a New Investigator Award from the Canadian Institutes of Health Research, and is the Medical Director and Chair in Implementation Science at the Peter Gilgan Centre for Women's Cancers at WCH in partnership with the Canadian Cancer Society. She was recently the recipient of the 2021 Canadian Cancer Society Inclusive Excellence Award. Her research program focusses on improving quality of care in cancer screening and prevention, particularly for populations that experience marginalization, through a variety of methods including community-partnered approaches.

Panelists

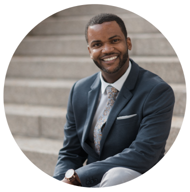

Dr. Andrew Thomas
President of the Black Physicians' Association of Ontario; Family Physician - Bowmanville Health Centre

Dr. Andrew Thomas is a Family Physician and President of the Black Physicians' Association of Ontario (BPAO). He is originally from Whitby, Ontario and then attended Howard University for Medical School. His interest in health promotion/disease prevention grew when he completed a Master's Degree in Public Health at the University of Toronto. He has a Family Practice in Durham Region where he teaches 3rd year Medical Students from Queen's University.

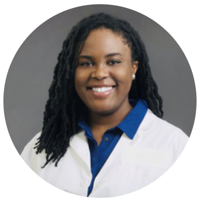

Dr. April Swoboda
Medical Oncologist and Assistant Professor of Oncology

April Swoboda, MD, is an academic medical oncologist. After graduating from Phillips Academy Andover in 2002, she enrolled in the Program in Liberal Medical Education (PLME) at Brown University, where she received her Bachelor's of Science in Neuroscience (2006) and her medical doctorate (2010). Dr. Swoboda completed Internal Medicine residency in 2013, then worked as oncology hospitalist for a year. She completed her Medical Oncology fellowship at McGaw Medical Center/Northwestern University in 2016, then pursued additional research training. Her research interests include triple-negative breast cancer, immunotherapy and the gut microbiome.

PANEL 1

Chantal Sorhaindo
Nurse Practitioner at the Department of Family and Community Medicine at the University of Toronto Family Practice Unit - St. Michael's Hospital

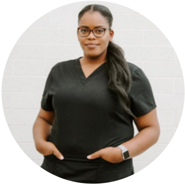

Chantal is an adjunct lecturer at the Lawrence S. Bloomberg Faculty of Nursing, UofT, and Session Tutor for the Temerty Faculty of Medicine at the UofT. She prides herself in engaging in anti-Black racism work and is the Chair of the Nurse Practitioner's Association of Ontario NPAO Black Nurse Practitioners' Community of Practice and is co-chair of the St. Michael's Hospital Department of Family and Community Medicine Anti-Black Anti-Indigenous racism Advisory Group. She is the co-chair of the UofT Lawrence S. Bloomberg Faculty of Nursing Equity, Diversity, Inclusion, Indigenous reconciliation committee, and she currently sits on the NPAO Board of Directors as Director, Primary Health Care.

Elysia Bryan
Ambassador for The Olive Branch of Hope

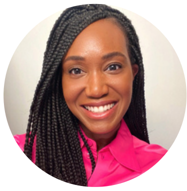

Elysia Bryan is a passionate advocate for breast health in young women. She was diagnosed with breast cancer at the age of 34 and learned that she carries the BRCA2 gene mutation. Her experience inspired her to become an ambassador with the Olive Branch of Hope, an organization dedicated to raising awareness and providing support to black women affected by breast cancer. Elysia's personal journey has taught her the importance of early detection and prevention, and she is committed to empowering women to take charge of their health and well-being.

Dr. Juliet Daniel
Cancer Biologist and the Associate Dean of Research and External Relations in the Faculty of Science at McMaster University

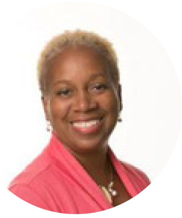

Professor Juliet Daniel received her BSc from Queen's University, her PhD from UBC, and completed Postdoctoral studies at St Jude Children's Research Hospital and Vanderbilt University in Tennessee. Prof. Daniel's research is focused on elucidating the role of the transcription factor Kaiso in cancer and vertebrate development. Her team is also currently elucidating the molecular/genetic causes of the disparities in incidence and poor outcomes of triple negative breast cancer in Black women. In recognition of her research and community service, Prof. Daniel has received several awards including the inaugural Canadian Cancer Society Inclusive Excellence Award, an Honorary Doctor of Science from the University of the West Indies (UWI) Cave Hill, a UWI Vice Chancellor's Award, and a WXN Canada's Most Powerful Women: Top 100 Award among many others.

BEST HEALTH FOR BLACK WOMEN 2023

PANEL 1

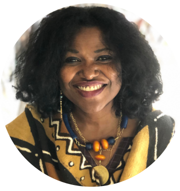

Dr. Nazik Hammad
Medical Oncologist at Saint Michael Hospital; and professor of medical oncology for the Department of Medicine; University of Toronto

Dr. Hammad's disease site interests are breast and gastrointestinal cancers. Her academic work and research interests include medical education and workforce development in low and middle-income countries (LMIC) particularly in sub-Saharan Africa and competency-based medical education in Canada and LMIC. She is currently co-chair of the Black Physicians Association of Ontario's (BPAO) Network for Advancing Black Medical Learners in Ontario (N-ABL). Her areas of education methodological expertise include training program development and evaluation, accreditation, curriculum development, mentoring, faculty development and education innovation. Other research and academic interests include value-based cancer care, global and local inequities and disparities in cancer, global health and global oncology and women as healthcare professionals. Together with colleagues in Africa she led the first Choosing Wisely Africa initiative. She is a co-author in the Lancet Oncology commission for Cancer in Sub-Saharan Africa and is a commissioner in the Lancet Commission on Women and Cancer.

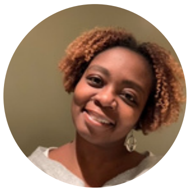

Laura Moore
Uncovered Project 2020 - Rethink Breast Cancer

Laura Moore was diagnosed in May of 2018, with stage 3, triple-negative breast Cancer - 18+ weeks of chemotherapy, one surgery consisting of a lumpectomy and axillary lymph node dissection, an additional eight months of chemotherapy and then 30 rounds of radiation. Her cancer journey was long and intense but she stands here today stronger and more resilient than she ever could have imagined. "I am so incredibly blessed and filled with gratitude that I get to go back to my 'normal' life. Now, my goal is to grow from a survivor to a thriver, an ally and an advocate."

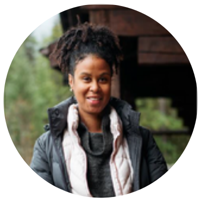

Michelle Audoin
Creator of the Uncovered Project, Key Collaborator at Rethink Breast Cancer

Michelle is a breast cancer advocate and community collaborator. She is the proud mother of two kids. Before being diagnosed with metastatic breast cancer in 2017, Michelle worked as a passionate educator for the Toronto District School Board. In 2020, she created a resource for the BIPOC community called Uncovered: A Breast Recognition Project, in collaboration with Rethink Breast Cancer. Michelle volunteers her time in various capacities at Rethink Breast Cancer and the Abbey Retreat Centre.

PANEL 2

GYNECOLOGICAL HEALTH

Moderated by:

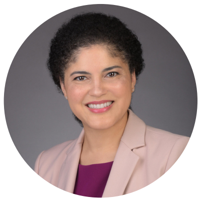

Dr. Cindy Maxwell
Vice President, Medical Affairs & System Transformation and Lead Medical Executive at Women's College Hospital

She is an accomplished physician, researcher and educator who has been recognized as a transformative health system leader. She is a Maternal Fetal Medicine Specialist at Mount Sinai Hospital and Women's College Hospital, and Professor, Faculty of Medicine, Department of Obstetrics and Gynaecology at the University of Toronto. Her clinical and research interests are focused on disorders of pregnancy with an emphasis on pregnancies affected by obesity, malignancy, and gastrointestinal disorders. Dr. Maxwell co-leads N-ABL, the provincial network to support Black medical learners and is a past-President of the Black Physicians Association of Ontario. Dr. Maxwell leads the Obesity Stream of the FIGO Committee on Impact of Pregnancy and Long-term Health. She is a Member of the Governing Council of the Provincial Council on Maternal and Child Health for Ontario.

Panelists

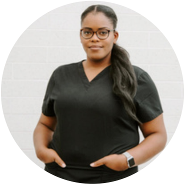

Chantal Sorhaindo
Nurse Practitioner at the Department of Family and Community Medicine, for the University of Toronto Family Practice Unit; St. Michael's Hospital

Chantal is an adjunct lecturer at the Lawrence S. Bloomberg Faculty of Nursing, UofT, and Session Tutor for the Temmetry Faculty of Medicine at the UofT. She prides herself in engaging in anti-Black racism work and is the Chair of the Nurse Practitioner's Association of Ontario NPAO Black Nurse Practitioners' Community of Practice and is co-chair of the St. Michael's Hospital Department of Family and Community Medicine Anti-Black Anti-Indigenous racism Advisory Group. She is the co-chair of the UofT Lawrence S. Bloomberg Faculty of Nursing Equity, Diversity, Inclusion, Indigenous reconciliation committee, and she currently sits on the NPAO Board of Directors as Director - Primary Health Care.

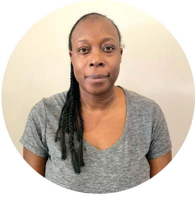

Cristel Cuffy
Canadian Forces Military Veteran; Founder of Tabono & Art

Cristel Cuffy is a first generation Canadian of parents born in Trinidad and Tobago. She is a Canadian Forces military veteran, having received a Ceremonial Decoration and was a recipient of the Queen Elizabeth II's Golden Jubilee Medal. She is a current police member whom is currently off with permanent disabilities sustained from her decades of service from both her careers combined. But regardless of her physical limitations, she has prioritized and embraced her spiritual, mental, and emotional growth as well as her passion for painting. Now she is the founder of Tabono & Art, where is offers large scale, custom, textured wall paintings that you can actually feel and fall in love with.

PANEL 2

Dr. Crystal Clark, Scientist and Associate Head of Research, Department of Psychiatry, Women's College Hospital; Associate Professor, Department of Psychiatry, University of Toronto

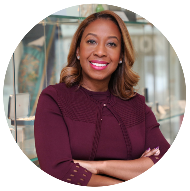

Crystal T. Clark, MD, MSc is a psychiatrist specializing in mood and anxiety disorders and is internationally recognized for her expertise in the treatment of women's mental health across the reproductive life span (i.e., menstruation, pregnancy, postpartum, infertility, trauma related to reproduction). Dr. Clark is the psychiatry research and clinical lead for perinatal bipolar disorder and Black maternal mental health at WCH. In her efforts to increase equity she is committed to the studies to characterize, develop, and increase prevention intervention strategies that address Black perinatal mental health.

Dr. Clark is a past president of the Marcé of North America and serves on the board of directors of Marcé International. Dr. Clark received her Bachelor of Arts in Psychology from Northwestern University and her Master of Science and Medical Doctorate from the University of Louisville, Kentucky (2006). She completed her adult psychiatry training and served as chief resident at Johns Hopkins in Baltimore, Maryland, USA (2010). She completed fellowship training in clinical research and reproductive psychiatry while also serving on faculty at the Baylor College of Medicine/Michael E. Debakey VA as well as the University of Pittsburgh Medical Center/ Pittsburgh VA Healthcare Systems. Prior to joining University of Toronto, she served as an Associate Professor, Fellowship Director, and Associate Program Residency Director at Northwestern University, Chicago.

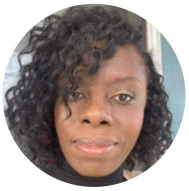

Jaime Sterling

Coming soon

PANEL 2

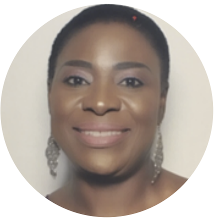

Dr. Modupe Tunde-Byass
President, Black Physicians of Canada; Associate Professor, University of Toronto; Staff Obstetrician and Gynecologist, North York General; Co-founder of Women's Health Education Made Simple (WHEMS)

Dr. Modupe Tunde-Byass is a Fellow of the Royal College of Obstetricians and Gynecologists of the UK and The Royal College of Surgeons of Canada. She is involved in key quality initiatives like increasing access to vaginal birth after cesarean section and improving the care of women undergoing early pregnancy complications and losses. As the President of the Black Physicians of Canada, she collaborates with national organizations like the Royal College of Physicians and Surgeons of Canada, Canadian Medical, Protection Association, Canadian Medical Association, Society of Obstetricians and Gynaecologists (to mention a few) in reviewing internal practices and policies related to Equity, Diversity, and inclusivity; additionally, she is involved with research around COVID -19 hesitancy and COVID-19 online mis/disinformation in Black communities. She is a co-author on healthcare management publication on IDEA as a leadership competency.

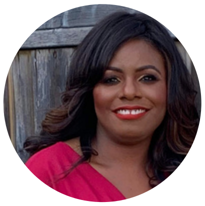

Sharon Cumberbatch
Supervisor with Toronto Employment and Social Services

Sharon Cumberbatch is a supervisor with Toronto Employment and Social Services where she coordinates system level planning to address the needs of vulnerable populations and equity deserving groups based on the social determinants of health. She forges key partnerships with non-profit organizations, government agencies, employers and the community. In 2010, she was the recipient of the General Manager's Award for Community Engagement. Sharon leads her office's Confronting Anti-Black Racism committee. Through this work, she identifies policies and develops strategies of accountability to address racism experienced by staff, clients and the community at large.

BEST HEALTH FOR BLACK WOMEN 2023

CLOSING SESSION

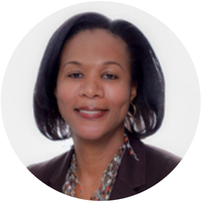

Elaine Goulbourne, Clinical Director, Primary Care & Peter Gilgan Centre for Women's Cancers/Director, Clinical Resources and Performance

Thank you !

RESOURCES

THE PETER GILGAN CENTRE FOR WOMEN'S CANCERS

No matter where you live in Canada

The Peter Gilgan Centre for Women's Cancers at Women's College Hospital gives **every woman every chance to access the highest standard of cancer care**.

For more information please visit:

https://bit.ly/WCHPeterGilganCentre

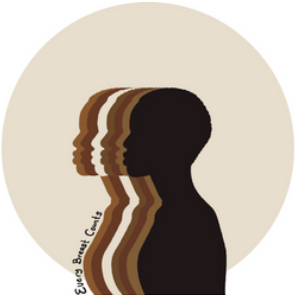

Every Breast Counts

A resource hub developed by **BLACK WOMEN FOR BLACK WOMEN**

Please visit:

https://bit.ly/EveryBreastCounts

The Olive Branch of Hope
Cancer Support Services

Don't Suffer in Silence
Join Our Community Care Family

No Matter where you are in Canada or the Caribbean

The Olive Branch of Hope is committed to making your breast cancer journey easier through our Sharing-Supporting & Surviving programs and offering you a safe place to share what **MATTERS to YOU**.

WE UNDERSTAND - We Care

Let's get connected

@hopeolivebranch
@theolivebranchofhope
www.theolivebranch.ca
@olivebranchofhope
olivebranch@theolivebranch.ca
416-256-3155

RESOURCES

Seeking more information, support and community

Learn more information about **Uncovered: A Breast Recognition Project**

www.rethinkbreastcancer.com/uncovered

Join the Rethink Network

A Safe, private space to connect with others who get what you're going through at:

www.rethinkbreastcancer.com/network

Stay connected!

@rethinkbreastcancer
@rethinkbreastcancer
@rethinktweet
hello@rethinkbreastcancer.com
www.rethinkbreastcancer.com

Have questions about breast cancer?

Contact the Cancer Information Helpline

info@cancer.ca
1-888-939-3333
cancer.ca

Do you need help finding information about cancer?

Visit the Community Services Locator at

cancer.ca/csl

Questions are answered by professionals who can provide evidence-based information and support.

BEST HEALTH FOR BLACK WOMEN 2023

THIS EVENT IS POSSIBLE THANKS TO:

THE PLANNING COMMITTEE:

- Dr. Aisha Lofters
- Amanda Etty
- Dr. Cindy Maxell
- Dawn Barker
- Donavan Miller
- Elaine Goulbourne
- Jasmine Sikand
- Laura Burnett
- Laura Moore
- Leila Springer
- Melessa Carlson
- Nicole Bourgeois
- Patricia Rabel
- Suzanne Charles Watson

OUR PARTNERS:

The Peter Gilgan Centre for Women's Cancers

The Olive Branch of Hope Cancer Support Services

rethink Breast Cancer

Canadian Cancer Society

THE MODERATORS AND SPEAKERS

MAIN SESSION

- Dr. Aisha Lofters
- Dawn Barker
- Janelle Griffith
- J. Nadia Headley

PANEL 1

- Dr. Aisha Lofters
- Dr. Andrew Thomas
- Dr. April Swoboda
- Chantal Sorhaindo
- *Elysia Bryan*
- *Dr. Juliet Daniel*
- *Dr. Nazik Hammad*
- *Laura Moore*
- *Michelle Audoin*

PANEL 2

- Dr. Cindy Maxwell
- *Chantal Sorhaindo*
- *Cristel Cuffy*
- *Dr. Crystal Clark*
- *Jaime Sterling*
- *Dr. Modupe Tunde-Byass*
- *Sharon Cumberbatch*

THANK YOU
